# Real-world use of maralixibat in biliary atresia: a case series

**DOI:** 10.3389/fped.2026.1858862

**Published:** 2026-08-14

**Authors:** Natasha Dilwali, Douglas B. Mogul, Shannon M. Vandriel, Tony Tokman, Catherine A. Chapin, Bertrand Roquelaure, Rossitsa Rousseva, Johanna Ferreira, Mercedes Martinez, Benno Kohlmaier

**Affiliations:** 1 Division of Pediatric Gastroenterology, Hepatology, and Nutrition, Johns Hopkins University, Baltimore, MD, United States; 2 Division of Pediatric Gastroenterology, Hepatology, and Nutrition, Columbia University Irving Medical Center, New York, NY, United States; 3 Mirum Pharmaceuticals, Inc., Foster City, CA, United States; 4 Department of Pediatrics, Ann & Robert H. Lurie Children’s Hospital of Chicago, Chicago, IL, United States; 5 APHM, Multidisciplinary Pediatrics Department, Timone Children’s Hospital, Marseille, France; 6 King’s College Hospital, London, United Kingdom; 7 Division of Pediatric Gastroenterology, Liver Disease and Nutrition, Cohen Children’s Medical Center, New Hyde Park, NY, United States; 8 Pediatric Gastroenterology, Hepatology, and Nutrition, University of Miami Miller School of Medicine, Miami, FL, United States; 9 Department of Pediatrics, Columbia University Irving Medical Center, New York, NY, United States; 10 Department of Pediatrics, Medizinische Universität Graz, Graz, Austria

**Keywords:** case report, cholestasis, IBAT inhibitor, liver transplant, pruritus

## Abstract

Biliary atresia (BA) is the most common cause of cholestasis in infants, and pruritus may be a prominent and debilitating complication. Maralixibat is the first US Food and Drug Administration–approved drug for the treatment of cholestatic pruritus in children with Alagille syndrome and has been subsequently approved for treatment of progressive familial intrahepatic cholestasis as well. Several patients with BA have received maralixibat as part of a compassionate use program. We report the use of maralixibat for the treatment of cholestatic pruritus in BA for six patients. Demographics, past medical history, laboratory markers, and medications were collected. Pruritus was assessed using the Clinician Scratch Scale (CSS) at baseline and last clinical follow-up. All patients reported improved CSS with no increased usage of other antipruritic medications, and the medication was well tolerated.

## Introduction

Biliary atresia (BA) is a progressive, obliterative disorder affecting varying lengths of both intra- and extrahepatic bile ducts, leading to fibrosis and cirrhosis in most patients. In the United States, BA occurs in approximately one in 10,000 to 20,000 births per year, making it the most common cause of cholestasis in infants and the leading indication for liver transplantation in all children (1–3). A Kasai portoenterostomy (KPE) remains the primary treatment strategy by attempting to reestablish bile flow and improve cholestasis (2). Despite KPE, many patients may have progression of cholestatic liver disease, developing complications such as scleral icterus, jaundice, acholic stools, failure to thrive, and pruritus (4). Decreased bile flow after KPE and associated elevated bile acids is thought to contribute to pruritus (4). Furthermore, existing literature on other cholestatic disorders has shown that pruritus significantly impacts quality of life (5–8).

Maralixibat is an ileal bile acid transporter (IBAT) inhibitor that blocks the reuptake of bile acids in the intestines and promotes fecal bile acid excretion. In 2021, maralixibat became the first drug approved by the US Food and Drug Administration for the treatment of cholestatic pruritus in children with Alagille syndrome (ALGS), and more recently, progressive familial intrahepatic cholestasis (PFIC) (9). Clinical trials have demonstrated improvement in pruritus as well as cholestasis biomarkers, such as bile acids and bilirubin (10, 11). However, there are limited data regarding the impact of IBAT inhibitors on cholestatic pruritus in BA. In this case series, we report using maralixibat to treat cholestatic pruritus in BA for six patients who received the drug as part of the compassionate use program (CUP).

## Methods

### Study design

The medical records of pediatric patients diagnosed with BA and treated with maralixibat through the CUP were retrospectively reviewed from March to October 2024 from six tertiary referral hospitals. Patients were eligible for maralixibat under compassionate use if they had cholestatic pruritus that was not responsive to standard-of-care treatments (e.g., rifampicin, antihistamines) and met appropriate safety criteria (e.g., no imminent need for a liver transplant). Eligibility determinations were made by the treating physician at the time of drug initiation, recognizing that disease trajectory in BA may progress despite meeting safety criteria at baseline. Patients were prescribed daily doses appropriate for their weight, which could be increased to twice daily doses for ongoing pruritus, if tolerated (up to 300 µg/kg orally twice daily). All patients had a confirmed diagnosis of BA based on standard clinical, imaging, surgical, and/or histopathologic criteria. Additional genetic testing to assess alternative genetic or metabolic causes was not routinely performed in this cohort, as it is not part of standard clinical management. A standardized electronic clinical report was used to collect key clinical variables from treating physicians.

### Study definitions and variables

The following parameters were collected: demographic characteristics, past medical history, height and weight Z-scores, and antipruritic medications. Additionally, laboratory values were collected at baseline (last value before starting maralixibat) and last follow-up (last value at time of survey completion). Pruritus was assessed using the Clinician Scratch Scale (CSS), a physician-observed assessment tool developed by Whitington et al. (12) The CSS is measured on a 0 to 4 scale as follows: 0 = none; 1 = rubbing or mild scratching when undistracted; 2 = active scratching without evident skin abrasions; 3 = abrasions evident; and 4 = cutaneous mutilation, hemorrhage, and scarring. A clinically meaningful pruritus response was defined as ≥1-point reduction in CSS score from treatment initiation to final clinical follow-up. Pruritus severity, assessed clinically using the CSS, was selected as the primary outcome rather than concurrent biochemical markers of cholestasis, as these markers do not reliably correlate with pruritus severity. Data were censored at survey completion (September 2024).

### Statistical analysis

Summary statistics for continuous variables were calculated, including median, minimum (min), and maximum (max). Categorical variables were calculated as frequencies/raw counts and percentages.

## Case description

The retrospective analysis included six pediatric patients (83.0% female) diagnosed with BA and treated with maralixibat. The median (min, max) age at KPE was 61 (33, 132) days. No patients had syndromic BA. One patient was undergoing liver transplant evaluation.

The median (min, max) age at first presentation of pruritus was 7.5 (3, 20) months. The median (min, max) age of maralixibat treatment initiation was 2.6 (0.4, 5.6) years. Immediately before initiating treatment, four patients (67%) had a CSS score of 3, and two patients (33%) had a CSS score of 4. At the time of treatment, all patients were prescribed at least two medications for management, most commonly ursodeoxycholic acid (disease modifying therapy with secondary benefit for pruritus) and rifampicin (antipruritic agent), which were both used in all patients.

**Figure 1:**
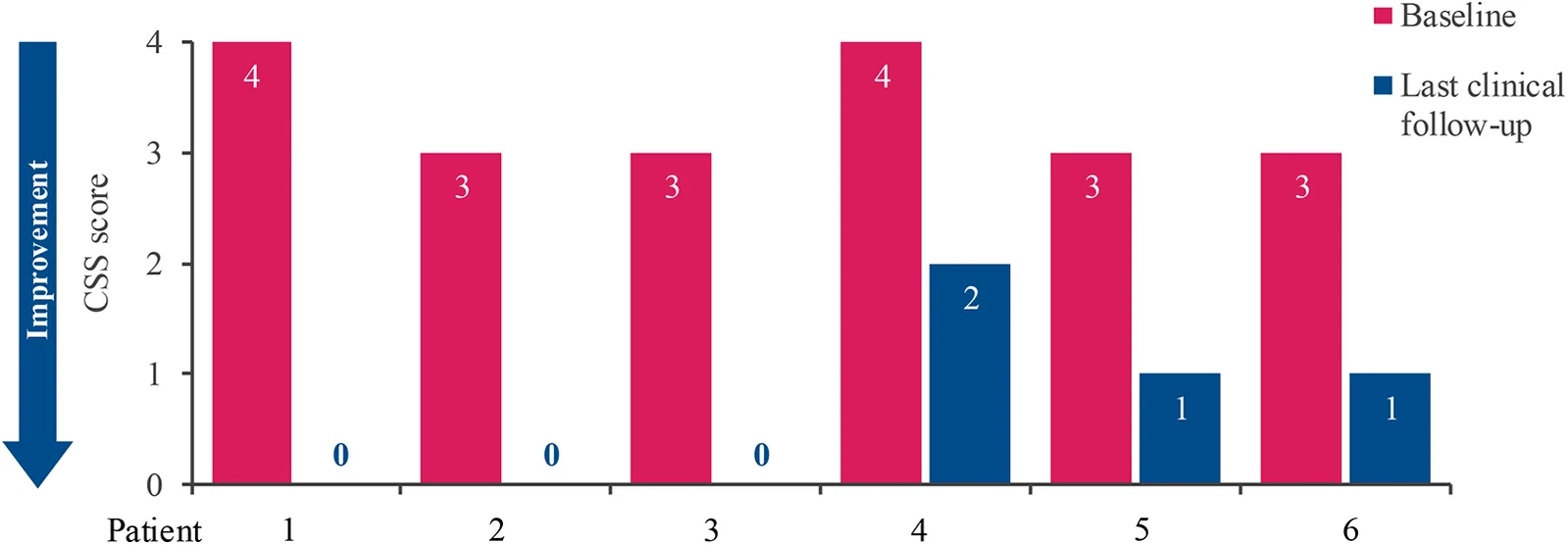
CSS scores at baseline and last clinical follow-up. CSS is a five-point scale, where 0 = none; 1 = rubbing or mild scratching when undistracted; 2 = active scratching without abrasions; 3 = abrasions; and 4 = cutaneous mutilations, hemorrhage, scarring. CSS, Clinician Scratch Scale.

After dose escalation, patients received maralixibat doses ranging from 284 µg/kg/d to 600 µg/kg/d orally. Patients received the medication for a median (min, max) of 7.5 (3, 13) months at the time of survey completion. During the treatment period, all six patients achieved a clinically meaningful pruritus response (≥1-point reduction in CSS score), and 3 patients (50%) achieved complete resolution of their pruritus (Figure 1). Two of the remaining patients decreased to a CSS score of 1, while the last patient decreased to a CSS of 2. Improvements in liver enzymes, bilirubin, serum bile acids, and platelets were inconsistently observed in these patients (Table 1). After initiation of maralixibat, three patients were able to decrease their overall antipruritic medication doses, and no patients had increased usage.

**Table 1:** Individual patient parameters at baseline and last clinical follow-up.

Parameter	Baseline (overall cohort)^a^	Patient 1		Patient 2		Patient 3		Patient 4		Patient 5		Patient 6	
		BL	FF	BL	FF	BL	FF	BL	FF	BL	FF	BL	FF
sBA, μmol/L	86 (22, 193)	86	16	188	131	76	34	193	87	22	10	401	159
Total bilirubin, mg/dL	1.0 (0.8, 9.5)	1.0	1.4	7.1	10.3	0.8	0.9	2.2	2.8	0.3	0.2	9.5	0.2
ALT, U/L	145 (23, 316)	109	142	156	340	316	174	133	132	23	23	210	38
AST, U/L	127 (38, 186)	98	127	159	236	186	126	127	129	38	38	139	40
GGT, U/L	199 (20, 639)	89	100	97	161	639	433	300	350	20	15	602	14
Height Z-score	−0.9 (−2.6, 0.2)^b^	0.2	3.0	−0.9	−0.7	n/a	1.9	−1.5	−1.4	0.9	−0.1	−2.6	−2.6
Weight Z-score	0.2 (−1.9, 1.4)	0.3	3.3	0.7	0.5	1.4	1.4	−0.7	−0.4	0.1	−0.2	−2.0	0.5

a All values are median (min, max) for all 6 patients unless otherwise specified.

b Based on five cases for which data were available. ALT, alanine aminotransferase; AST, aspartate aminotransferase; BL, baseline; FF, final follow-up; GGT, gamma-glutamyl transferase; n/a, not available; sBA, serum bile acids.

Two patients ultimately received liver transplants while on maralixibat. One patient (Patient 6) had history of splenomegaly, ascites requiring treatment with diuretics, growth failure, and fat-soluble vitamin deficiency before starting maralixibat, reflecting advanced liver disease at baseline. Similarly, a second patient (Patient 4) had history of splenomegaly, thrombocytopenia, and gastrointestinal bleeding before starting maralixibat and was listed for a liver transplantation nine months after initiating maralixibat. With respect to potential adverse events, one patient had mild abdominal pain and a transient transaminase elevation, which resolved on subsequent laboratory monitoring without the need for treatment interruption or changes in medication dosing. These findings were followed at routine visits and required no intervention. Another patient experienced three episodes of loose stools during the first week after treatment initiation. A second patient also developed diarrhea at treatment initiation. This patient was started on 190 µg/kg/d and was slowly titrated to the age-appropriate dosing without issue. The diarrhea in these two patients was manageable, and there were no additional clinical sequelae or dose modifications. Systematic longitudinal fat-soluble vitamin levels were not available for all patients during maralixibat therapy as part of this review, though deficiencies were present in some patients prior to treatment initiation.

## Discussion

This case series describes the use of maralixibat for the treatment of cholestatic pruritus in BA through a CUP. All patients treated with maralixibat had clinically meaningful improvements in pruritus, and three patients had complete resolution of pruritus. The drug appeared to be generally well tolerated in this small cohort, with the most common adverse event being diarrhea, which is expected given the mechanism of action. However, the diarrhea was manageable.

Although more commonly seen in patients with ALGS and PFIC, pruritus can occur in nearly all cholestatic liver diseases, including BA. Even after KPE, patients with BA can experience debilitating pruritus, which impacts quality of life and can be an indication for transplantation (13). Maralixibat inhibits the reuptake of bile acids in the distal ileum, thereby promoting their fecal excretion and decreasing the amount in circulation. The drug serves as a pharmacologic interruption of bile acid transport, based on the premise that surgical interruption of bile acid return successfully reduces pruritus in patients with ALGS and PFIC (14). Clinical trials in ALGS and PFIC have demonstrated improved cholestasis biomarkers and meaningful reduction in pruritus (10, 11). Similarly, in our case series, we observed a meaningful pruritus response in all patients. In addition, half of our patients were able to decrease their use of concomitant antipruritic medications, and no patients escalated antipruritic therapy. Although patients were deemed not to have an imminent need for liver transplantation at the time of maralixibat initiation, two patients progressed to transplant listing or transplantation during follow-up. Both patients had features of portal hypertension, growth failure, or fat-soluble vitamin deficiency prior to treatment initiation, underscoring the advanced disease present in this cohort. These outcomes were interpreted by the treating physicians as unrelated to maralixibat exposure but rather a reflection of disease severity and the natural history of BA. Consistent with previous trials, maralixibat was well tolerated, without significant complications causing discontinuation of the medication. Additional approved therapies for cholestatic pruritus in BA are necessary, as pruritus can significantly decrease quality of life for these patients. A limitation of our case series is that CSS is qualitative and subject to bias. An additional limitation is the lack of genetic testing in these patients. However, the clinical phenotype of BA is generally distinct from PFIC, and the diagnosis of BA includes findings by cholangiogram at the time of KPE (7, 13, 15). Further, this series lacks a control group to assess the true impact of treatment more rigorously. Although height and weight Z-scores were available at baseline and final follow-up, the small sample size and limited follow-up duration make it difficult to draw conclusions on growth trajectories. However, growth parameters remained stable throughout the treatment period, suggesting that maralixibat did not negatively affect growth in this cohort. Additionally, systematic evaluation of fat-soluble vitamin levels was not available for all patients and therefore, conclusions regarding long-term safety should be interpreted cautiously. There is a need for future large randomized controlled trials studying the effect of maralixibat on the treatment of pruritus in BA and other cholestatic disorders. This is a primary aim of the EXPAND trial (NCT06553768) (16).

## Conclusion

In this case series, we observed a meaningful pruritus response in all six patients, with three patients experiencing complete resolution. These results suggest that maralixibat may be a useful treatment option for patients with BA who have cholestatic pruritus.

## Data Availability

The original contributions presented in the study are included in the article/supplementary material, further inquiries can be directed to the corresponding author/s. Beginning six months and ending five years after publication, deidentified participant data might be made available to investigators whose proposed use of the data has been approved by a review committee, including the primary authors and the study funder. Proposals should be directed to grants@mirumpharma.com. Before being granted access, data requesters will be required to sign a data access agreement.
